# Sialome diversity of ticks revealed by RNAseq of single tick salivary glands

**DOI:** 10.1371/journal.pntd.0006410

**Published:** 2018-04-13

**Authors:** Jan Perner, Sára Kropáčková, Petr Kopáček, José M. C. Ribeiro

**Affiliations:** 1 Institute of Parasitology, Biology Centre of the Czech Academy of Sciences, České Budějovice, Czech Republic; 2 Faculty of Science, University of South Bohemia, České Budějovice, Czech Republic; 3 Laboratory of Malaria and Vector Research, National Institute of Allergy and Infectious Diseases, Bethesda MD, United States of America; Institute of Tropical Medicine Antwerp, BELGIUM

## Abstract

Ticks salivate while feeding on their hosts. Saliva helps blood feeding through host anti-hemostatic and immunomodulatory components. Previous transcriptomic and proteomic studies revealed the complexity of tick saliva, comprising hundreds of polypeptides grouped in several multi-genic families such as lipocalins, Kunitz-domain containing peptides, metalloproteases, basic tail secreted proteins, and several other families uniquely found in ticks. These studies also revealed that the composition of saliva changes with time; expression of transcripts from the same family wax and wane as a function of feeding time. Here, we examined whether host immune factors could influence sialome switching by comparing sialomes of ticks fed naturally on a rabbit, to ticks artificially fed on defibrinated blood depleted of immune components. Previous studies were based on transcriptomes derived from pools of several individuals. To get an insight into the uniqueness of tick sialomes, we performed transcriptomic analyses of single salivary glands dissected from individual adult female *I*. *ricinus* ticks. Multivariate analysis identified 1,279 contigs differentially expressed as a function of time and/or feeding mode. Cluster analysis of these contigs revealed nine clusters of differentially expressed genes, four of which appeared consistently across several replicates, but five clusters were idiosyncratic, pointing to the uniqueness of sialomes in individual ticks. The disclosure of tick quantum sialomes reveals the unique salivary composition produced by individual ticks as they switch their sialomes throughout the blood meal, a possible mechanism of immune evasion.

## Author summary

In this work, we confirm previous reports that the repertoire of tick salivary gland transcripts changes as a function of time, but in addition, we now identify transcripts that change their levels according to the mode of feeding of ticks. Implementation of membrane feeding allowed us to feed ticks on an immune-deficient diet and identify transcripts that are subject to immunity-stimulated expression. Such identification may help to prioritise selection of salivary gland transcripts for further investigation. One novelty of this work was creating cDNA libraries from a single pair of salivary glands, which helped to gain insight into sialomic diversity at the single tick level. We observed that ticks express a battery of genes in defined clusters as feeding progresses (over tested replicates), but also individual ticks were found to express idiosyncratic clusters of genes. Such a biological phenomenon may imply novel tick mechanisms for evading host-mediated recognition of tick antigens.

## Introduction

Ticks remain attached to their hosts for several days or weeks while increasing their weight several hundred fold. The tick acquires its meal from a skin feeding cavity containing a haemorrhagic pool that continually supplies blood to the ectoparasite. This feeding pool is maintained by tick saliva that is intermittently secreted into the host while alternating sucking and spitting throughout the meal [1]. Tick saliva contains a complex potion of pharmacologically active compounds including non-peptidic components such as vasoactive and immunosuppressive prostaglandins and adenosine, as well as peptidic kratagonists (agonist binding proteins active against serotonin, histamine, inflammatory leukotrienes and cytokines), anti-clotting, anti-platelet, anti-complement and immunosuppressive peptides; metalloproteases can digest fibrin and inhibit angiogenesis [2, 3].

Hosts can mount immune responses against ticks and non-natural hosts can mount very effective anti-tick immunity [4]. Perhaps for this reason, tick salivary proteins evolve at a fast pace to avoid immune recognition. Many salivary components of *Ixodes* ticks are members of expanded multigene families, such as lipocalin, Kunitz, cystatin, basic tail, ixostatin, antigen-5, and metalloprotease families (for information on these families, see [2]). Interestingly, many members of these families are expressed only at a particular time during feeding [5–7]. In other words, the mechanism of paralogue switching may cause the host antibody-mediated response to fail because the antibody response takes several days, but by the time the antibody is produced, its target is gone and a new paralog is in place.

The sialome switching mechanism could theoretically be driven by a physiological feeding “clock”. Alternatively, the switch could be triggered by a host response, such as innate and/or acquired immunity or a tick “stressor” signal, or both. The information on tick sialomes (from the Greek sialo = saliva) has been so far assembled from conventional Sanger or next generation (454 or Illumina) transcriptomes, made from pools of dozens of salivary glands. This approach is informative for obtaining the general sialomic repertoire that a given population of a species of tick can produce but it is not informative about which components of this repertoire are being recruited by individual members of that population. In the present work, we made libraries from single adult female *I*. *ricinus* ticks that were fed for 24, 48, or 72 h, artificially, using membrane feeding, or naturally on a rabbit. The artificial feeding treatment was an attempt to investigate whether the lack of host inflammatory and innate immunity responses would suppress sialome changes. Moreover, this comparative approach led to the identification of several transcripts that are specifically up-regulated by the host immune response at an early stage of feeding, and therefore may have potential as candidates for effective anti-tick interventions. We identified an additional 434 novel polypeptide sequences within these 1,279 contigs, most from secreted products at 72 h of feeding, a time point not previously explored with *I*. *ricinus* sialo-transcriptomes [7, 8]. Nearly 2,000 novel sequences were identified and deposited in public databases.

## Methods

### Ethics statement

All animal experiments were carried out in accordance with the Animal Protection Law of the Czech Republic No. 246/1992 Sb, ethics approval No. 095/2012 and protocols approved by the responsible committee of the Institute of Parasitology, Biology Centre of the Czech Academy of Sciences.

### Ticks and laboratory animals

**Fig 1:**
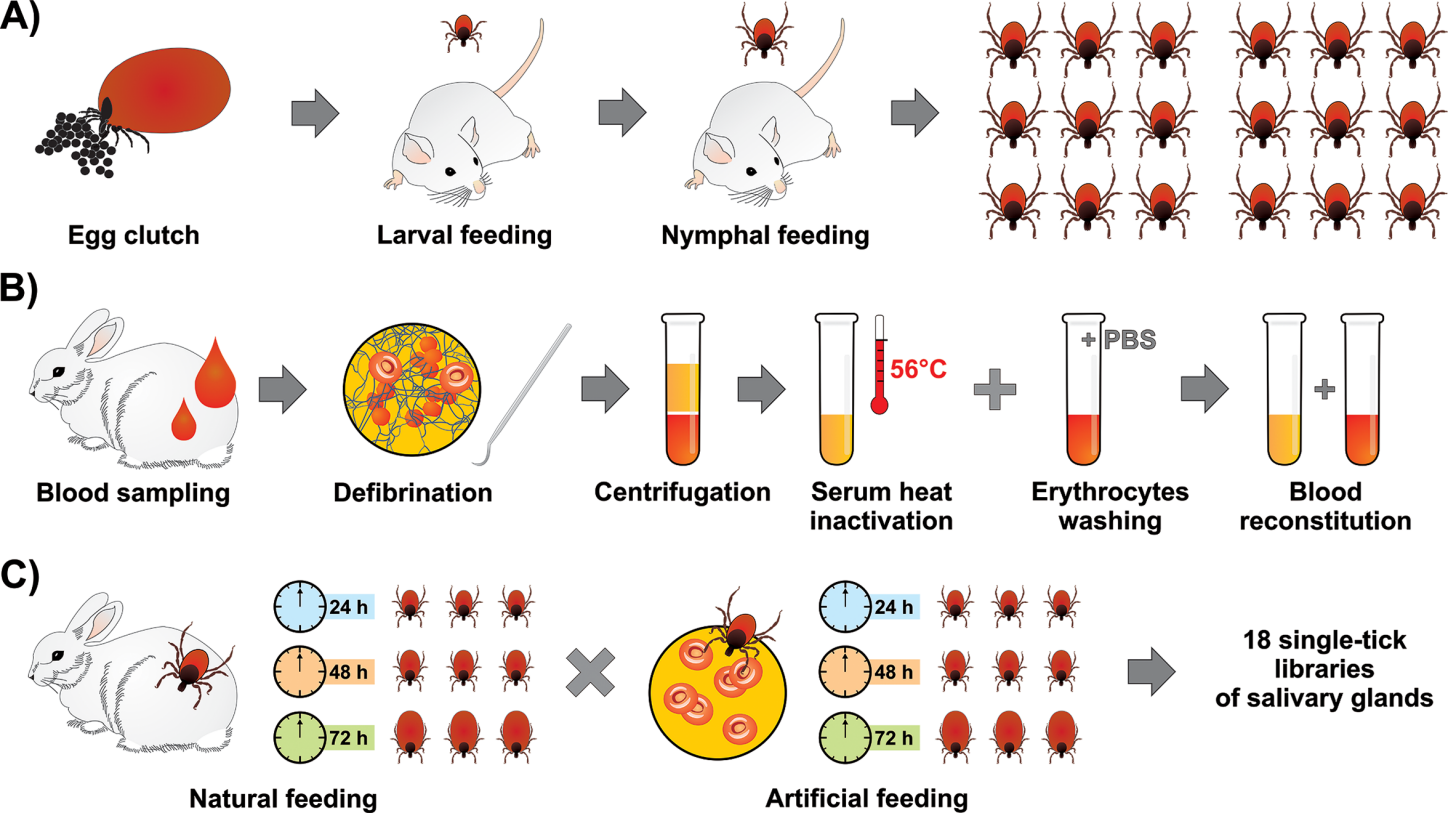
Schematic illustration of the experimental design. **A) Raising first-generation *Ixodes ricinus* adult siblings.** The *I*. *ricinus* female cohort used in the study originated from one egg clutch, with larvae and nymphs being fed on laboratory mice. **B) Preparation of blood meal for artificial membrane feeding.** A laboratory rabbit was bled and the collected blood was manually defibrinated. Blood was fractioned by centrifugation and the collected serum was heat inactivated at 56°C for 30 min in a water bath. Red blood cells were repeatedly washed in sterile PBS. Heat inactivated serum was then added to red blood cells to reconstitute the blood meal. **C) Scheme of library preparation.** Ticks were fed naturally on rabbit or artificially on reconstituted rabbit blood. Ticks were removed at three time-points 24 h, 48 h, and 72 h. From each tick, a pair of salivary glands was dissected and RNA was extracted. Each time-point and mode of feeding is represented by three individual ticks, i.e. three independent libraries. Overall, this study is based on 18 single-tick salivary gland libraries.

Male and female adult *Ixodes ricinus* ticks were collected by flagging in a forest near the town České Budějovice in the Czech Republic. Laboratory rabbits reared in the animal facility of the Institute of Parasitology were used for adult tick feeding. Females were allowed to oviposit. Larvae and derived nymphs were fed on guinea pigs. Adult ticks from a single egg batch were used for this work (**Fig 1**). Resulting adult female siblings were then fed either naturally on a rabbit or fed a reconstituted rabbit blood exploiting an artificial membrane feeding system (see below).

### Blood meal preparation

Rabbit blood was manually defibrinated using an autoclaved fork. The whole blood was then separated by centrifugation 2500 x g, 15 min, 4°C. Resulting serum was then transferred into a fresh tube and the red blood cells were washed 3 times in sterile PBS to remove remaining white cells (as evidenced by Giemsa staining). Collected serum was heat inactivated at 56°C for 30 min in 15-ml tubes using a water bath. The heat inactivated (HI) serum was then sterile filtered using a 0.22 μm filter (Merck Millipore). The efficiency of heat inactivation was verified as described [9]. Briefly, *Escherichia coli* (TOP10, Invitrogen) was grown to OD = 1 in Lysogeny Broth (LB), then diluted one thousand times to 10^6^CFU/ml, and 50 μl of the culture was mixed with 50 μl of: a) sterile PBS, b) gentamicin (10 μg/ml), c) active serum, d) heat inactivated serum. The cultures were then incubated at 225 rpm, 37°C for 3 h and then plated out on LB plates. To reconstitute the whole blood, the HI serum was then added back to washed red blood cells. Unaltered hematocrit levels were verified by SDS-PAGE (20μg protein per lane) and UV/VIS spectrophotometry (2μl drop of 100 times diluted blood meal), as previously described [10] (**S1 Fig**).

### Artificial membrane feeding

Artificial membrane feeding of *I*. *ricinus* females was performed according to Kröber and Guerin [11] using stationary feeding units as described previously [10, 12]. The feeding unit was lined with a thin (80‒120 μm) silicone membrane, treated with a bovine hair extract prepared in dichloromethane (0.5 mg of low volatile lipids), and UV-sterilised. Fifteen *I*. *ricinus* females were put in the assembled feeding unit and allowed to attach. After 8 hours, all unattached females were removed and an equal number of males to attached females were added into the feeding unit. Reconstituted rabbit blood was served without supplementation and was regularly exchanged in 8h intervals. At least three females of comparable sizes were then forcibly removed from the membrane after 24, 48, and 72 hours of feeding.

### Tick salivary glands (SG) preparation and total RNA extraction

Salivary glands were dissected from a single tick in biological triplicates for both treatments–rabbit-fed (R), membrane-fed (M), and three time points of feeding (24 h, 48 h and 72 h), giving in total 18 individual pairs of salivary glands (**Fig 1**). Ticks were dissected on a double sticky tape in a drop of DEPC-treated PBS. The purity of dissected SG was checked by microscope and all contaminating tissues, mainly trachea, were removed. The salivary glands were then homogenized in RA1 buffer (Machery Nagel) using a syringe and 29G needle. Total RNA was extracted using a Nucleospin RNA kit (Machery Nagel). The quality of the RNA samples was confirmed by lab-on-chip analysis using the 2100 Bioanalyzer (Agilent Technologies, Inc. Santa Clara, CA, USA).

### Illumina sequencing

Total RNA samples (labelled according to the feeding mode and time as M24_1,2,3; R24_1,2,3; M48_1,2,3; R48_1,2,3; M72_1,2,3; R72_1,2,3) were submitted to the North Carolina State Genomic Sciences Laboratory (Raleigh, NC, USA) for Illumina RNA library construction and sequencing. Purification of messenger RNA (mRNA) was performed using the oligo-dT beads provided in the NEBNext Poly(A) mRNA Magnetic Isolation Module (New England Biolabs, USA). Complementary DNA (cDNA) libraries for Illumina sequencing were constructed using the NEBNext Ultra Directional RNA Library Prep Kit (NEB) and NEBNext Mulitplex Oligos for Illumina (NEB) using the manufacturer-specified protocol. Briefly, the mRNA was chemically fragmented and primed with random oligonucleotides for first strand cDNA synthesis. Second strand cDNA synthesis was then carried out with dUTPs to preserve strand orientation information. The double-stranded cDNA was then purified, end repaired and “a-tailed” for adaptor ligation. Following ligation, the samples were selected for a final library size (adapters included) of 400–550 bp using sequential AMPure XP bead isolation (Beckman Coulter, USA). Library enrichment was performed and specific indexes for each of the 18 samples were added during the protocol-specified PCR amplification. The amplified library fragments were purified and checked for quality and final concentration using an Agilent 2100 Bioanalyzer with a High Sensitivity DNA chip (Agilent Technologies, USA). The final quantified libraries were pooled in equimolar amounts for sequencing on four lanes of an Illumina HiSeq 2500 DNA sequencer, utilizing a 150 bp single end sequencing flow cell with a HiSeq Reagent Kit v4 (Illumina, USA). Flow cell cluster generation for the HiSeq2500 was performed using an automated cBot system (Illumina, USA). The software package Real Time Analysis (RTA), version 1.18.64, was used to generate raw bcl, or base call files, which were then de-multiplexed by sample into fastq files for data submission using bcl2fastq2 software version v2.16.0. The raw fastq files were deposited in the Sequence Read Archives (SRA) of the National Center Biotechnology Information (NCBI) under accession SRP071001 of bioproject PRJNA312361 and biosample SAMN04497582.

### Bioinformatics analysis

Assembly of all reads was done as described previously using the assemblers Abyss and Soapdenovo-Trans with every kmer ending in 1 and 5 (-k program switch) from 21 to 95 [13–17]. Resulting contigs were re-assembled by a pipeline of blastn and cap3 assembler [18] as described earlier [19]. Coding sequences were extracted based on blastx [20] results deriving from several database matches, including a subset of the non-redundant protein database of the NCBI containing tick and other invertebrate sequences, as well as the Swissprot and Gene Ontology (GO) databases. Open reading frames larger than 150 nt were also extracted if they had signal peptides indicative of secretion, as evaluated by version 3.0 of the SignalP program [21]. Reads from the four libraries were mapped back into the CDS by blastn with a word size of 25 and allowing one gap. Reads were mapped up to a maximum of five different CDS if the blast scores were the same for all matches. The program edgeR was used in ancova mode to detect statistically significant differentially expressed genes (DEG) according to treatment or time variables and displayed as Volcano plots [22]. EdgeR inputted the read matrix for genes having at least one library expressing a RPKM [23] (fragments per thousand nucleotides per million reads) equal or larger than 10. For heat map display [24] of the CDS temporal expression, Z scores of the RPKM values were used. Heatmaps were produced with the programs gplots and heatmap.2 using R [25]. Differential gene expression clustering was done with the program Expander version 7.1 [26], using as input, RPKM data and the click algorithm. More details of the input are available in the results section, which also contains hyperlinks to several databases, as explained previously [19, 27]. Deduced coding sequences and their translations were deposited to the Transcriptome Shotgun Assembly database DDBJ/EMBL/GenBank under the accessions GEGO01000001-GEGO01007692

### cDNA synthesis and RT-qPCR

cDNA preparations were made from 0.1 μg of total RNA using the Transcriptor High-Fidelity cDNA Synthesis Kit (Roche Diagnostics, Germany). The cDNA served as templates for subsequent quantitative expression analyses by RT-qPCR. Samples were analysed by a LightCycler 480 (Roche) using Fast Start Universal SYBR Green Master Kit (Roche). Relative expressions were calculated by the ΔΔCt method. The expression profiles were normalised to *I*. *ricinus* elongation factor 1α (ef-1α). Primers used for validation are listed in **S1 Table**.

## Results and discussion

### Preparation of single tick-libraries of salivary glands

The feasibility of the recently introduced technique for artificial feeding of hard ticks [11] allowed us to investigate the as yet insoluble problem of identification of biologically active components from tick saliva that specifically react to the host’s immune/inflammatory response. In order to address the question, how do ticks avoid active host defense mechanisms, we compared the sialomes of individual ticks fed at different times on a natural host to the sialomes of ticks fed artificially on a blood meal deprived of active host immunity components. The deprived natural immunity was produced by serum heat inactivation and washing out of the remaining white cells from manually defibrinated blood (**Fig 1** and **S1 Fig**). Adult *I*. *ricinus* females that were used in this study were derived from a single egg batch to reduce genetic variation. Our experimental design consisted of two feeding modes: (a) natural feeding on a laboratory rabbit and (b) artificial feeding of heat-inactivated rabbit blood. Individual pairs of salivary glands were dissected at three time points (24, 48 and 72 h) with 3 replicates for each time-point, thus needing the construction of 18 libraries (**S2 Table**). The “*de novo*” assembly of these 18 tick transcriptomes enabled the identification of 1,907 novel protein sequences, 406 of which were classified as of a secreted nature. We additionally extended 115 sequences that were at least 95% identical to publicly available proteins, but 5% or longer in length. Previous *I*. *ricinus* sialo-transcriptomes were assembled from over half a billion Illumina and pyrosequencing reads [5, 7, 8], but they did not include 72h samples from adult ticks, from whence the majority of these new transcripts were derived, emphasizing the still unknown dimension of the full sialome repertoire of *I*. *ricinus*.

### Transcriptomes of tick salivary glands change in response to mode of feeding and feeding progression

**Fig 2:**
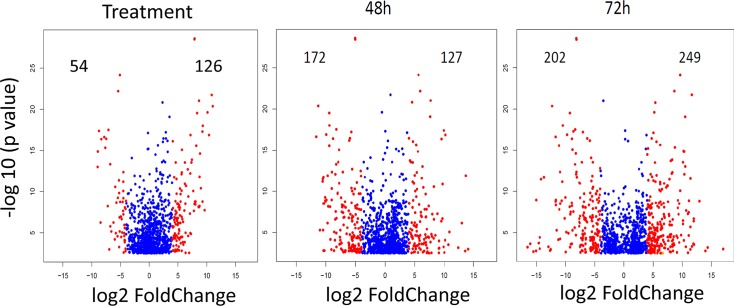
Volcano plots representing the differentially expressed *Ixodes ricinus* salivary expressed contigs according to feeding treatment (rabbit or membrane) and time of feeding (48 or 72 h). The number of differentially expressed contigs (FDR < 0.05) expressed in the salivary glands of *I*. *ricinus* having a log_2_ fold change (log_2_FC) higher than 4 or smaller than -4 according to feeding treatment (rabbit or membrane) and time of feeding at 48 or 72 hours, after analysis of covariance by edgeR with intercept at 24 h. The red dots represent contigs with log_2_ fold change larger than 4 or smaller than -4. The numbers indicate the count values for these contigs. The analysis was done with the edgeR analysis of covariance model [22].

These 18 libraries were loaded in each of three Illumina HiSeq 2500 lanes (single ended protocol), obtaining from 18.5 to 27 million reads for each library, averaging 149‒150 nt in length with a median and L50 size of 151 after removal of contaminating primers (**S2 Table**). The ~435 million reads were assembled together with the previously assembled salivary and midgut transcriptome of *I*. *ricinus* [5, 12], from which 40,490 coding sequences (CDS) were extracted. All deducted coding sequences and their reads are available for browsing as a hyperlinked Excel spreadsheet (further referred to as Source data 1) at http://exon.niaid.nih.gov/transcriptome/Ixric-18/S1-web.xlsx. After mapping the reads of the 18 libraries to these CDS and calculating the RPKM for each, 20,773 contigs contained at least one library yielding a RPKM equal or larger than 10 (Source data 1). To identify differentially expressed genes (DEG), we submitted the read matrix of these 20,773 contigs to an analysis of covariance (ANCOVA) test using the edgeR package. A total of 1,279 contigs were identified as being differentially expressed with a false discovery rate (FDR) < 0.05. The results of the ANCOVA can be found in columns FW‒GG of the Source data 1 spreadsheet, and DEG’s for feeding treatment and times of feeding can be identified by sorting the columns for the respective fold change. Volcano plots (**Fig 2**) show the large degree of variation found, with ranges of expression being over 2^10^. These uncommonly large rates have been detected previously in tick salivary transcriptomes as a function of time [5, 6]. Selected up-regulated transcripts from 48 h to 24 h and from 72 h to 48 h of naturally-fed ticks are listed in **S2** and **S3 Tables**, respectively. Most abundant transcripts across replicated libraries of each time-point of naturally-fed ticks are listed in **S5**, **S6**, and **S7 Tables**.

### Cluster identification among DEGs

**Fig 3:**
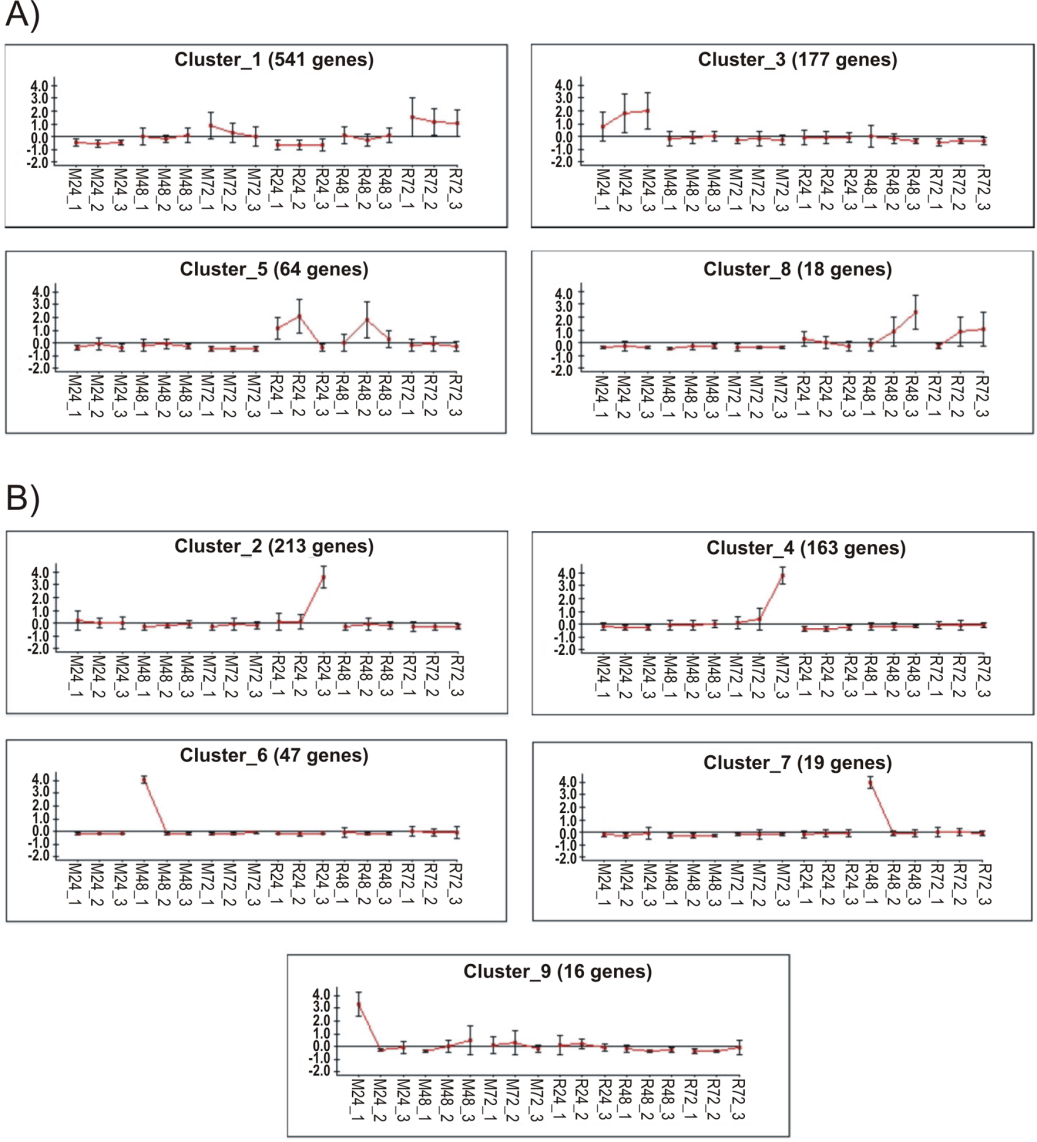
Clusterization of differentially expressed salivary genes of *Ixodes ricinus* according to type (membrane or rabbit) and time (24, 48 and 72 h). Clusterization was done with the click algorithm [28] of the expander software [26]. A) Clusters associated with type and/or time of feeding. B) Idiosyncratic clusters. The Y axis represents the Z normalized expression levels.

**Fig 4:**
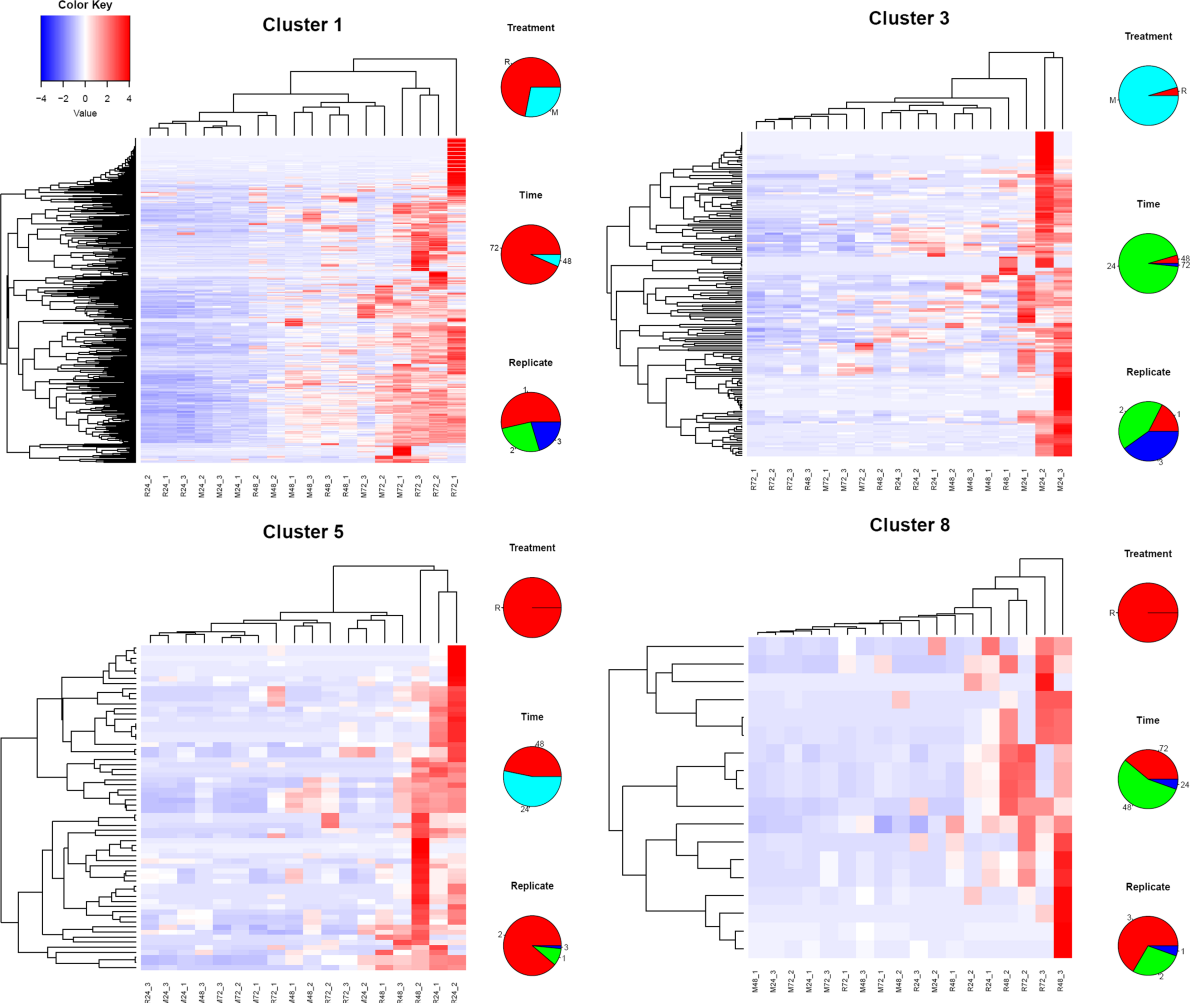
Heat maps of the clusters of genes indicated in Fig 3A. The pie charts on the right of each heat map represent the frequencies of feeding treatment (top), time (center) and replicate number (bottom) of the contig producing the highest RPKM value. The color key represents the color code of the Z scores.

We hypothesised that the absence of active host immunity would lead to a stable sialome, without much time change in the M as opposed to the R mode of feeding. Indeed the ANCOVA revealed the mode significance, suggesting this hypothesis to be true. However, the M group also showed time-dependent differential expression. To obtain further insight into the sialo-transcriptome DEG’s, we submitted the RPKM data for all 1,279 contigs identified as DEGs by edgeR for cluster analysis using the click algorithm of the Expander package [26, 28]. Following Z normalization of the data and using default parameters, nine clusters were identified, leaving out 21 singletons. Column X of the Source data 1 spreadsheet contains the cluster membership number of the contigs. Sorting on this column can retrieve the contigs for each cluster. Of the nine clusters, four were clearly related to time and/or type of feeding (**Fig 3A**). Cluster 1 has 541 genes over-represented in both M and R types at 72 h, representing a time dependent/feeding mode-independent group of genes. Cluster 3 with 177 genes is M 24h over-represented; clusters 5 and 8 are both over-represented in rabbit feeding at 24 h / 48h and 48 h / 72 h, respectively. Clusters 5 and 8, specifically, may represent groups of genes responsive to host immune/inflammatory interaction. The over-representation was not always observed in all three replicates. **Fig 4** depicts the heat maps of these clusters. For each contig, the sample producing the larger RPKM value was recorded regarding type of feeding (M or R), time of feeding (24, 48, or 72 h), and replicate number (1, 2, or 3).

### Idiosyncratic single-tick clusters

**Fig 5:**
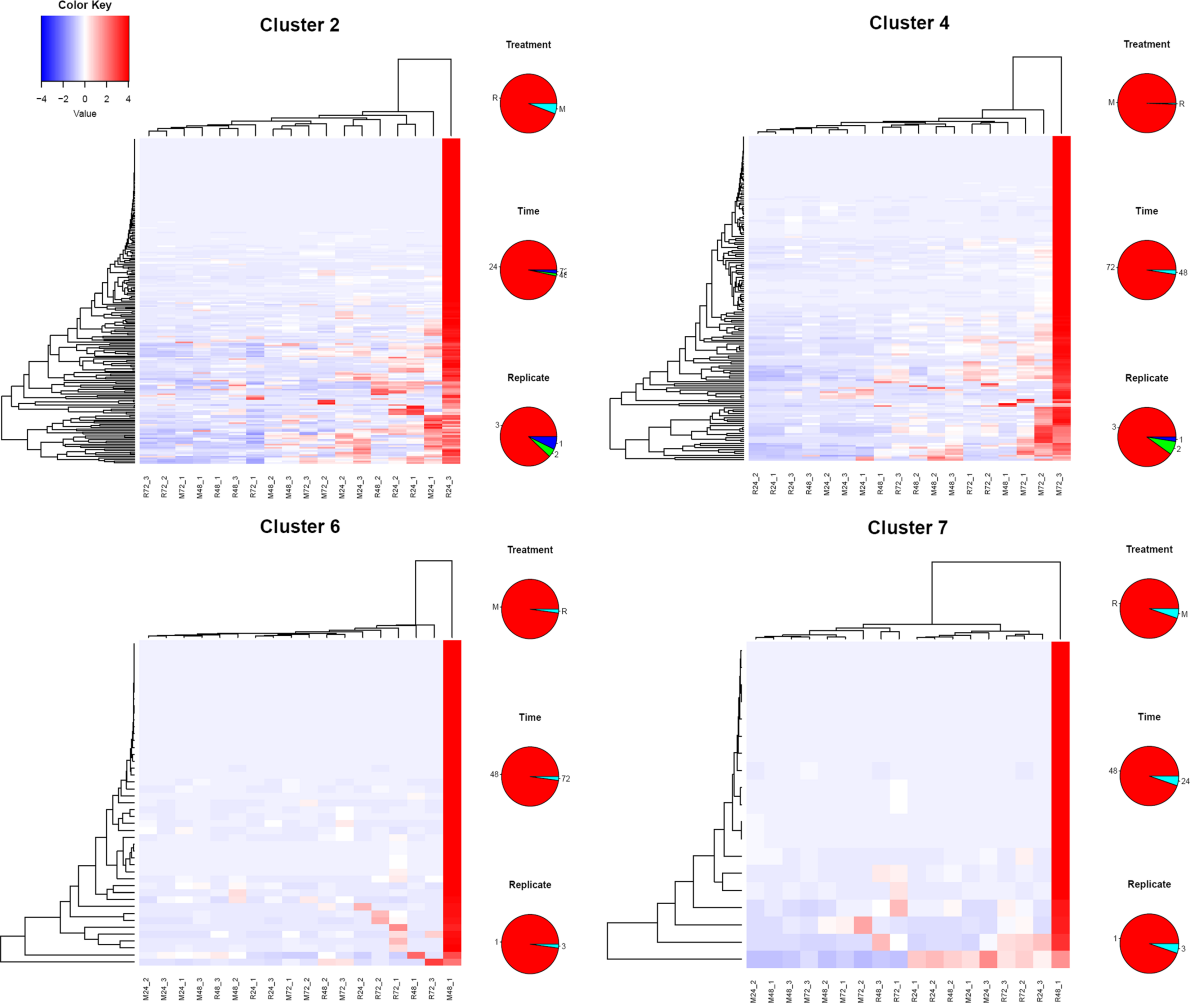
Heat maps of four of the five idiosyncratic clusters of genes indicated in Fig 3B. The pie charts on the right of each heat map represent the frequencies of feeding treatment (top), time (center) and replicate number (bottom) of the contig producing the highest RPKM value. The color key represents the color code of the Z scores.

While four of the nine clusters were associated with time or mode of feeding of a particular set of ticks, five of the nine clusters had a single tick responsible for the cluster of DEG’s (**Fig 3B**). Heat maps of four of the idiosyncratic clusters shown in **Fig 5** display their unique gene expression pattern. The majority of these idiosyncratic DEGs were of the secreted class, and included classic salivary members of the Kunitz, lipocalin, basic tail, and 8.9 kDa. For each contig, the sample producing the larger RPKM value was recorded regarding type of feeding (M or R), time of feeding (24, 48, or 72 h), and replicate number (1, 2, or 3). Since five of nine clusters of DEGs were ascribed to single ticks (**Fig 3B**) and since the majority of the encoded proteins in these clusters are secreted proteins, these results indicate that five of the 18 ticks were secreting a unique sialome repertoire. This may indicate that sialomes switch more frequently than the 24 h intervals that were used in this study. These idiosyncratic sialomes could represent, for example, “typical” average sialomes of 36 h or 60 h, not sampled in this study.

Even though there is a growing body of data that suggests tight time-dependent regulation of gene expression in salivary glands of feeding ticks, virtually nothing is known about the actual mechanisms that orchestrate the individual sialome switches. We have identified multiple transcripts encoding histone-modifying enzymes that were suggested to manage changes in sialome diversity [5]. Histone acetylases of GNAT (Ir-271878) and MYST (Ir-260682, Ir-242196, Ir-256281) families were moderately expressed (RPKM values range spans between 4‒33) across all libraries. Histone deacetylases (Ir-240628, Ir-261552, Ir-963, Ir-262316) were expressed with RPKM values ranging between 2‒37 across all libraries. We have also identified two transcripts encoding bromodomain proteins (Ir-248264 and Ir-250997), which are expressed with RPKM values ranging between 4‒16 across all libraries. Surprisingly, we have identified around forty contigs encoding lysine methyltransferases, but only a single arginine methyltransferase (Ir-262971) and single histone demethylase (Ir-239028). All of these transcripts, however, displayed very stable expression levels with very low inter-individual variability, and it is therefore difficult to conclude that these enzymes would orchestrate sialome switching. Tick salivary glands also express numerous copies of general transcription factor-encoding transcripts. Since approximately half of the assembled transcripts encode secretory proteins, not only timed orchestration of gene transcription, but also appropriate protein synthesis and folding must be organised. A transcript (Ir-258585) encoding X-box binding protein 1 (XBP-1) was found as the most abundant transcription factor across our libraries. This gene encodes a homologue of *Drosophila melanogaster* “bZIP”-containing transcription factor, which participates in the unfolded protein response, an evolutionarily conserved signalling pathway activated by an overload of misfolded proteins in the endoplasmic reticulum [29]. This transcription factor was implicated in the biology of the fly salivary glands, where the protein was suggested to stimulate the folding capacities of the ER in cells committed to intense secretory activities [30]. This transcription factor might, therefore, switch on a battery of proteins, as a stress response, to facilitate correct folding of thousands of proteins in tick salivary glands.

### Identification and verification of SG transcripts up-regulated by natural feeding

**Fig 6:**
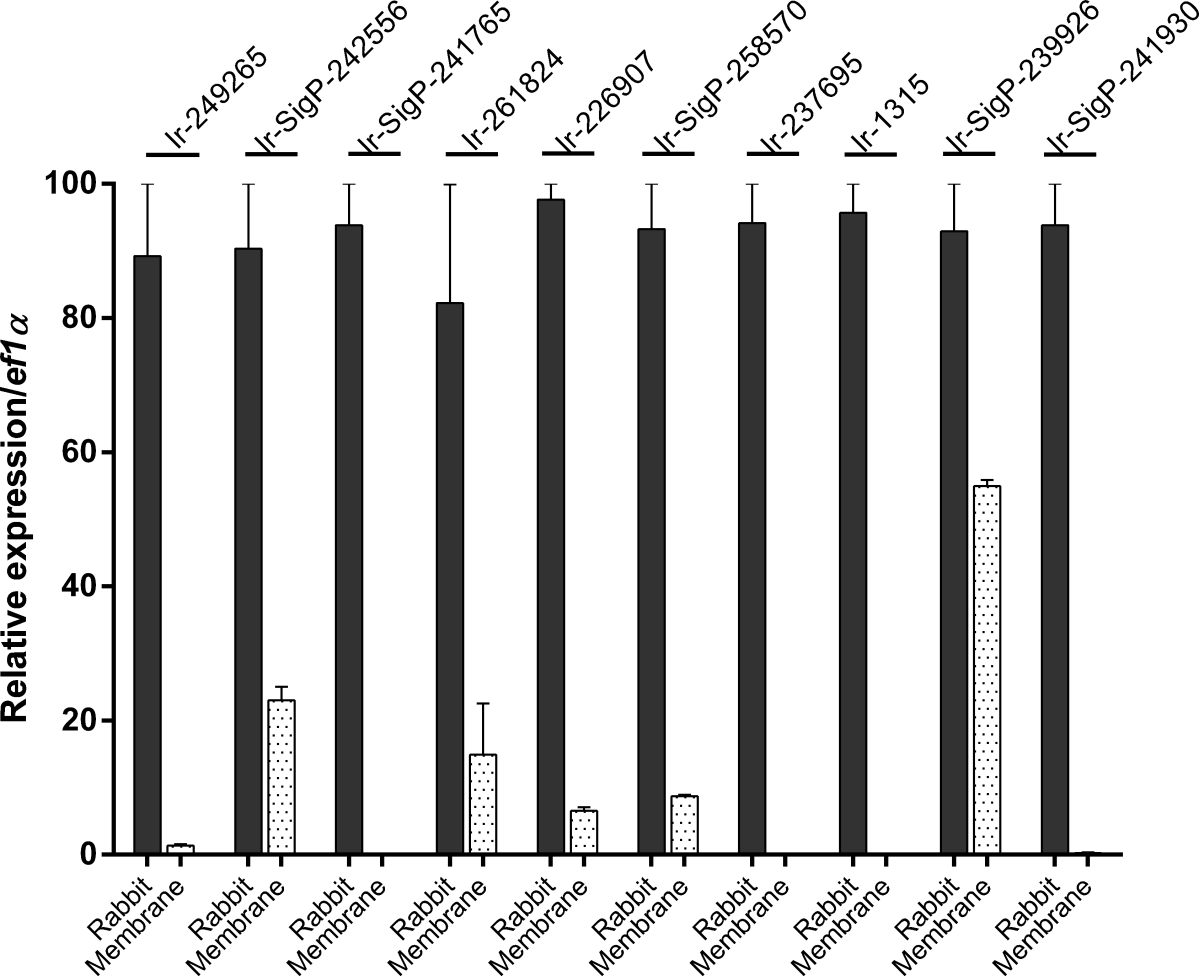
RT-qPCR analyses of differentially expressed transcripts in the tick SG dissected from *I*. *ricinus* females at 24 h. The melting curve analysis was verified and data were normalised to elongation factor 1 (*ef1α*). Mean and SEM are shown, n = 3 (biological replicates). For annotation of the transcripts, see the Table 1 or the Source data 1 spreadsheet at http://exon.niaid.nih.gov/transcriptome/Ixric-18/S1-web.xlsx.

Feeding treatment and time both promoted significant differentially expressed transcripts. However, the mode of feeding yielded a narrower range of differential expression and a smaller number of DEGs in comparison to time variables. We have identified individual transcripts with statistically significantly up-regulated levels in libraries of artificially fed ticks in comparison to naturally fed ticks. The transcripts are listed in **Table 1** for the 24 h time-point. To support our DEG analysis, we ran RT-qPCR analyses on cDNA templates from an independent tick cohort. We selected 24 h libraries, as this time interval seems to be the most relevant for potential targeting of tick-borne pathogen transmission. We have confirmed elevated levels of most identified DEGs in libraries of naturally fed ticks (**Fig 6**). These transcripts encode metalloproteases (Ir-249265, Ir-226907, Ir-237695), 18.3 kDa basic tail superfamily proteins (Ir-SigP-242556, Ir-SigP-258570), anti-complement proteins (Ir-SigP-241930; Ir-261824, Ir-SigP-239926), and secreted protein precursor (Ir-1315). To rebut the argument that membrane fed ticks underwent slower feeding progression and, therefore, did not reach the peak levels of expression, we also verified transcript levels at 48 h and 72 h time-points. As the RPKM values of these transcripts in membrane-fed ticks did not increase, even at later time-points, we concluded that expression of the verified DEGs was indeed induced by active components of the host blood. DEGs upregulated in naturally fed ticks at 48 h and 72 h are listed in **S8** and **S9 Tables**, respectively.

**Table 1:** Overview of RKPM values for significantly up-regulated contigs in rabbit-fed (R) compared to membrane-fed (M) ticks fed for 24 hours. Three independent libraries (1‒3) were used for each mode of feeding. Transcripts were listed by fold change and were filtered with coverage > 50, average RPKM > 10, and fold change > 5.

Link to Pep	Comments	E value	Coverage %	M24_1 RPKM	M24_2 RPKM	M24_3 RPKM	R24_1 RPKM	R24_2 RPKM	R24_3 RPKM
Ir-SigP-219629	18.3 kda subfamily of the Basic tail superfamily	3,0E-55	99,3	**0,4**	**0,2**	**0,2**	**2695,6**	**987,3**	**2594,7**
Ir-SigP-242556	18.3 kda subfamily of the Basic tail superfamily	2,0E-59	100	**3,2**	**2,4**	**2,1**	**17011,5**	**6302,5**	**15365,9**
Ir-SigP-241930	anticomplement protein 1	0,0E+00	100	**0,0**	**0,1**	**0,0**	**2377,0**	**2011,7**	**375,2**
Ir-261824	anticomplement protein 2	9,0E-92	91,5	**0,0**	**0,1**	**0,1**	**91,5**	**32,2**	**91,2**
Ir-SigP-229700	Ticks ixostatin	3,0E-17	103,1	**0,0**	**0,0**	**0,1**	**32,3**	**17,6**	**8,6**
Ir-SigP-258570	18.3 kda subfamily of the Basic tail superfamily	1,0E-48	100	**3,6**	**3,2**	**2,9**	**430,8**	**166,1**	**510,8**
Ir-SigP-241765	antigen 5 protein—signalP detected	2,0E-90	86	**2,4**	**6,4**	**0,2**	**77,2**	**202,6**	**141,6**
Ir-1315	secreted protein precursor	0,0E+00	85,6	**2,2**	**6,0**	**0,1**	**98,1**	**60,6**	**34,9**
Ir-369	Secreted metalloprotease	0,0E+00	95,7	**44,9**	**0,0**	**5,2**	**156,2**	**132,5**	**296,7**
Ir-SigP-369	Secreted metalloprotease	0,0E+00	100	**48,8**	**0,1**	**7,2**	**172,3**	**145,1**	**319,6**
Ir-249265	Secreted metalloprotease	0,0E+00	100	**31,2**	**13,4**	**8,6**	**175,6**	**125,4**	**87,7**
Ir-226907	Secreted metalloprotease	0,0E+00	85,2	**4,9**	**0,1**	**5,7**	**20,1**	**15,9**	**33,0**
Ir-SigP-239926	anticomplement protein IxAC-B5 precursor	8,0E-67	70,3	**337,0**	**87,9**	**109,7**	**1444,0**	**1074,8**	**479,2**
Ir-237695	Secreted metalloprotease	0,0E+00	89,8	**9,7**	**0,1**	**12,3**	**34,2**	**29,0**	**57,8**
Ir-249264	Secreted metalloprotease	0,0E+00	76,5	**7,7**	**20,5**	**1,9**	**47,5**	**36,3**	**66,8**

Our results show that three out of sixteen contigs encoding members of the 18.3 kDa subfamily of basic tail superfamily (use alphabetical sorting in column AB of the Source data 1 spreadsheet) are clearly responsive to active host immunity compared to membrane feeding (Table 1). On the other hand, contig Ir-SigP-255938 was substantially expressed in early M(24)-fed compared to R(24)-fed ticks and the other 18.3kDa members displayed quite inconsistent inter-individual variability (Source data 1). Some of the other 54 basic-tail proteins encoding transcripts (tagged as BTSP in column AB of the Source data 1 spreadsheet) were highly expressed independent of the feeding mode during all feeding intervals and belong to the most abundant transcripts during the 48 and 72 hours post attachment (**S6** and **S7 Tables**, respectively). These results accord well with previously published results on expression of BTSP superfamily members in *I*. *ricinus* females [7] and collectively suggest an important and specific role of these molecules at the tick-host interface, which is still not well understood and definitely worthy of further investigation.

One of the largest multigene families found in our transcriptome project comprises 173 contigs annotated as secreted metalloproteases (Column AB of Source data 1 spreadsheet), five of those belonging to the most host-responsive transcripts at the early stage of feeding (**Table 1**). These enzymes are believed to function as anti-hemostatic and anti-angiogenic factors [31, 32] and their expression in *I*. *ricinus* salivary glands was reported to be specifically dependent on the stage and feeding status [5].

Of special interest are three transcripts encoding host-responsive *I*. *ricinus* anti-complement proteins at the early stage of feeding (**Table 1**). Anti-complement activity of tick saliva was discovered three decades ago in *I*. *scapularis* (formerly *I*. *dammini*) [33] and one molecule responsible for this activity, tagged as Isac (for *I*. *scapularis* salivary anti-complement) was purified and cloned [34]. The sialome analysis of *I*. *scapularis* later revealed the existence of a multigene family encoding Isac paralogs in this species [32]. In our transcriptome (Source data 1) we found 10 contigs encoding proteins homologous or closely related to the previously characterized Isacs from *I*. *ricinus*, namely IRAC I and IRAC II [35], *Ix*AC B1‒5 [36], or other putative Isac anti-complement proteins identified more recently in the *I*. *ricinus* salivary gland transcriptome [7]. IRAC I and II as well as *Ix*AC B1‒B5 proteins were reported to inhibit the alternative pathway of mammalian complement activation via binding to properdin that prevents the formation of the C3/FactorB convertase complex [36]. The sequential expression of individual Isac functional homologs with divergent primary structures (antigenic variability) illustrates how ticks are capable of avoiding the host antigen-specific immune response in the course of feeding [37]. Since the vertebrate complement system apparently plays a decisive role in susceptibility or resistance to infection by *Borrelia burgdorferi* sensu lato [38–40], an effective targeting of anti-complement activities in tick saliva holds promise as a possible strategy to prevent the transmission of Lyme disease.

The tick salivary gland is a complex tissue that facilitates fluid absorption and secretion when off- or on-host, respectively [41]. As ticks feed, the tissue undergoes structural changes to facilitate secretion of bioactive components of saliva [42]. Previous studies revealed that feeding progression is linked with a timed regulation of gene expression in several tick species [5, 43, 44]. These studies clearly demonstrated that mRNA transcript repertoires differ substantially among groups of pooled salivary glands dissected at different time-points of feeding. In this study, we evaluated salivary gland transcriptomes from an “individualised” point of view and described single tick sialome responsiveness to active host immune components at the initial time-points of feeding. The list of all assembled contigs and their respective RPKM values are available through a hyper-linked spreadsheet: http://exon.niaid.nih.gov/transcriptome/Ixric-18/S1-web.xlsx, and can be blasted using a TSA blast, BioProject number 312361. The disclosure of tick quantum sialomes reveals the unique salivary composition of individual ticks as they switch their sialomes throughout the blood meal. The idiosyncratic nature of several quantum sialomes suggests sialomes switch within less than 24 h, the sampling time of this study. These switches have been proposed to exist as a mechanism of immune evasion [5–7, 32] but we now can start to appreciate their diversity and individual tempos. The rapid changes in transcript repertoires beg the following questions to be addressed because interference with the mechanism of sialome switching may become an interesting target for tick and tick-borne pathogen control: What is the mechanism underlying sialome switching? Does it involve classical transcription factor activation/suppression activated by a signal transduction cascade? Is it subject to epigenetic regulation?

Moreover, we demonstrated that artificial membrane feeding of hard ticks enables the identification of salivary gland transcriptome responses to dietary stimuli. Comparing sialomes at given time-points, but differing in the mode of blood-meal status (immunologically-passive blood meal in artificial feeding versus immunologically-active blood meal in natural feeding), we identified several transcripts that were expressed only upon feeding on active blood-meal, i.e. naturally-fed on a host (**Fig 2, Table 1**). Anti-complement proteins, metalloproteases, the 18.3 kDa basic-tail protein superfamily, and a secreted protein precursor were substantially up-regulated in salivary glands of naturally-fed ticks at 24 h, a time-point essential for tick attachment and pathogen transmission. These data thus reveal a novel list of potential vaccine candidates that are inducibly expressed and might assist in evading host immunity and/or facilitate pathogen transmission.

## Supporting information

S1 Fig Preparation of blood meal deprived of active host immunity components. (**A**) Verification of serum heat inactivation using cultured *Escherichia coli*. LB plates are shown. (**B**) Removal of leukocytes from red blood cells by repeated washing with sterile PBS. Giemsa-stained blood smear is shown. Scale bar indicates 10 μm. (**C**) Reconstitution of the heat inactivated serum with washed red blood cells to the original haemoglobin concentration verified by SDS-PAGE and VIS spectrophotometry. For details, see Material and Methods.
(TIF)

S1 Table Oligonucleotides used for RT-qPCR verification. (DOCX)

S2 Table Summary of reads resulting from Illumina sequencing of 18 salivary gland libraries from individual adult female *Ixodes ricinus* ticks feeding artificially or on a rabbit for 24, 48, and 72 hours. (DOCX)

S3 Table Overview of RKPM values for significantly up-regulated contigs in rabbit-fed (R) ticks fed for 48 h compared to 24 h. Three independent libraries (1‒3) were used for each time-point.
(DOCX)

S4 Table Overview of RKPM values for significantly up-regulated contigs in rabbit-fed (R) ticks fed for 72 h compared 48 h. Three independent libraries (1‒3) were used for each time-point.
(DOCX)

S5 Table Overview of RKPM values for most abundant transcripts in 24 h libraries of naturally-fed ticks. Three independent libraries (1‒3) were used.
(DOCX)

S6 Table Overview of RKPM values for most abundant transcripts in 48 h libraries of naturally-fed ticks. Three independent libraries (1‒3) were used.
(DOCX)

S7 Table Overview of RKPM values for most abundant transcripts in 72 h libraries of naturally-fed ticks. Three independent libraries (1‒3) were used.
(DOCX)

S8 Table Overview of RKPM values for significantly up-regulated contigs in rabbit-fed (R) compared to membrane-fed (M) ticks fed for 48 hours. Three independent libraries (1‒3) were used for each mode of feeding. Transcripts were listed by statistical significance and were filtered with coverage > 50, average RPKM > 10, and fold change > 5.
(DOCX)

S9 Table Overview of RKPM values for significantly up-regulated contigs in rabbit-fed (R) compared to membrane-fed (M) ticks fed for 72 hours. Three independent libraries (1‒3) were used for each mode of feeding. Transcripts were listed by statistical significance and were filtered with coverage > 50, average RPKM > 10, and fold change > 5.
(DOCX)
