# Supplementary figures and images for: Sialome diversity of ticks revealed by RNAseq of single tick salivary glands
Source: PLoS Negl Trop Dis. 2018 Apr 13;12(4):e0006410. doi: 10.1371/journal.pntd.0006410 (PMC5919021; doi:10.1371/journal.pntd.0006410)

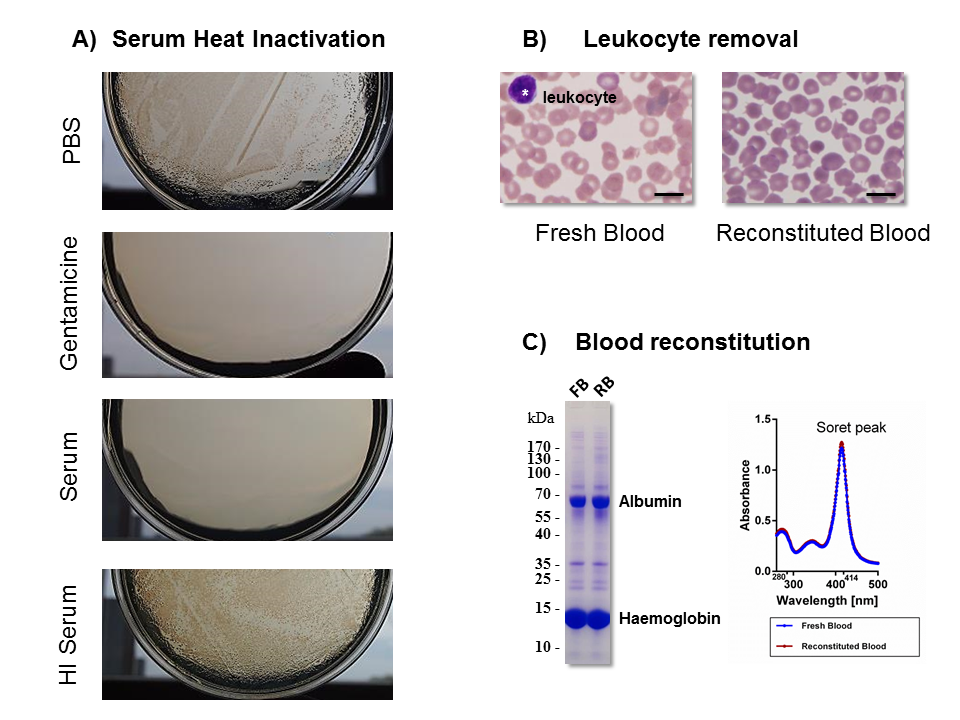

Supplement: S1 Fig — (A) Verification of serum heat inactivation using cultured Escherichia coli. LB plates are shown. (B) Removal of leukocytes from red blood cells by repeated washing with sterile PBS. Giemsa-stained blood smear is shown. Scale bar indicates 10 μm. (C) Reconstitution of the heat inactivated serum with washed red blood cells to the original haemoglobin concentration verified by SDS-PAGE and VIS spectrophotometry. For details, see Material and Methods. (TIF) [file pntd.0006410.s001.tif]
