# Supplementary material for: Sialome diversity of ticks revealed by RNAseq of single tick salivary glands
Source: PLoS Negl Trop Dis. 2018 Apr 13;12(4):e0006410. doi: 10.1371/journal.pntd.0006410 (PMC5919021; doi:10.1371/journal.pntd.0006410)
Supplement: S1 Table — (DOCX) [file pntd.0006410.s002.docx]

**S1Table.**  **Oligonucleotides used for RT-qPCR verification**

| **Contig / gene** | **Forward primer 5’-3’** | **Reverse primer 5’-3’** | **Amplicon length** |
| --- | --- | --- | --- |
| Ir-249265 | CGAGGCAAATATGTTCACTGG | CGCTCTTTAGTGGACCATCACC | 95 bp |
| Ir-SigP-242556 | GGATATTTCTACGGGATCTAC | TATAACACCAACCACTTCTG | 105 bp |
| Ir-261824 | CGAAGAGTCTCGTCCTGTG | CAGTTATATTCGCCGTGC | 78 bp |
| Ir-226907 | AACGGCGATCATAAAGTTCC | GCAAGCAGATGAGCATTGTG | 73 bp |
| Ir-SigP-258570 | GACGATGTGTTCCACGAAC | GTTGTGCAAGGGAACTTG | 56 bp |
| Ir-237695 | AAGCGCGGCTTTGCAACTG | CTCGAGAGCGCGTTCTTG | 147 bp |
| Ir-1315 | ATGCCTGATATGGCGGAG | TTTGGTTTCGGCCAGAAC | 55 bp |
| Ir-241765 | GCTCACTTTACACAGTTG | ATACTGCGTAGCCACATC | 61 bp |
| Ir-SigP-239926 | GTGAACAAGACATGGGAGGG | AGTTGCACATCAACCCAAC | 76 bp |
| Ir-SigP-241930 | ACAGAGCACAAGCAAAGGTG | ATATTCGCCGTGCATTCTTC | 105 bp |
| ef-1α | ACGAGGCTCTGACGGAAG | CACGACGCAACTCCTTCAC | 81 bp |
