# Supplementary material for: Sialome diversity of ticks revealed by RNAseq of single tick salivary glands
Source: PLoS Negl Trop Dis. 2018 Apr 13;12(4):e0006410. doi: 10.1371/journal.pntd.0006410 (PMC5919021; doi:10.1371/journal.pntd.0006410)
Supplement: S2 Table — (DOCX) [file pntd.0006410.s003.docx]

**S2 Table.**  **Summary of reads resulting from Illumina sequencing of 18 salivary gland libraries from individual adult female *Ixodes ricinus* ticks feeding artificially or on a rabbit for 24, 48, and 72 hours.**

| **Library name** | **Membrane or Rabbit fed** | **Time (h)** | **Total number of sequences** | **Total number of residues** | **Average length** | **Median size** | **L50** |
| --- | --- | --- | --- | --- | --- | --- | --- |
| M24_1 | Membrane | 24 | 26,648,382 | 3,994,219,581 | 149.89 | 151 | 151 |
| M24_2 | Membrane | 24 | 19,550,865 | 2,924,196,885 | 149.57 | 151 | 151 |
| M24_3 | Membrane | 24 | 25,265,096 | 3,782,418,745 | 149.71 | 151 | 151 |
| M48_1 | Membrane | 48 | 21,092,293 | 3,147,417,945 | 149.22 | 151 | 151 |
| M48_2 | Membrane | 48 | 22,670,024 | 3,389,271,656 | 149.50 | 151 | 151 |
| M48_3 | Membrane | 48 | 23,861,549 | 3,575,063,622 | 149.83 | 151 | 151 |
| M72_1 | Membrane | 72 | 24,125,461 | 3,614,381,790 | 149.82 | 151 | 151 |
| M72_2 | Membrane | 72 | 29,348,534 | 4,400,440,425 | 149.94 | 151 | 151 |
| M72_3 | Membrane | 72 | 23,035,466 | 3,456,438,984 | 150.05 | 151 | 151 |
| R24_1 | Rabbit | 24 | 18,662,674 | 2,793,280,598 | 149.67 | 151 | 151 |
| R24_2 | Rabbit | 24 | 19,734,696 | 2,952,549,483 | 149.61 | 151 | 151 |
| R24_3 | Rabbit | 24 | 26,474,265 | 3,955,650,986 | 149.41 | 151 | 151 |
| R48_1 | Rabbit | 48 | 28,204,630 | 4,210,673,875 | 149.29 | 151 | 151 |
| R48_2 | Rabbit | 48 | 24,500,679 | 3,671,757,864 | 149.86 | 151 | 151 |
| R48_3 | Rabbit | 48 | 23,476,841 | 3,504,508,487 | 149.28 | 151 | 151 |
| R72_1 | Rabbit | 72 | 25,481,071 | 3,806,435,511 | 149.38 | 151 | 151 |
| R72_2 | Rabbit | 72 | 26,034,181 | 3,889,347,225 | 149.39 | 151 | 151 |
| R72_3 | Rabbit | 72 | 27,265,628 | 4,077,636,150 | 149.55 | 151 | 151 |
|  |  |  |  |  |  |  |  |
| **Total** |  |  | 435,432,335 | 65,145,689,812 |  |  |  |
