# Supplementary material for: Sialome diversity of ticks revealed by RNAseq of single tick salivary glands
Source: PLoS Negl Trop Dis. 2018 Apr 13;12(4):e0006410. doi: 10.1371/journal.pntd.0006410 (PMC5919021; doi:10.1371/journal.pntd.0006410)
Supplement: S3 Table — Three independent libraries (1‒3) were used for each time-point. (DOCX) [file pntd.0006410.s004.docx]

**S3 Table.**  **Overview of RKPM values for significantly up-regulated contigs in rabbit-fed (R) ticks fed for 48 h compared to 24 h.** Three independent libraries (1‒3) were used for each time-point.

| **Link to Pep** | **Comments** | **E value** | **Coverage %** | R24_1 RPKM | R24_2 RPKM | R24_3 RPKM | R48_1 RPKM | R48_2 RPKM | R48_3 RPKM |
| --- | --- | --- | --- | --- | --- | --- | --- | --- | --- |
| Ir-SigP-83258 | salivary kunitz domain protein | 4,0E-24 | 100 | **0,0** | **0,7** | **0,0** | **8,0** | **9,6** | **8,5** |
| Ir-240295 | vesicular amine transporter | 0,0E+00 | 100 | **1,3** | **0,7** | **0,3** | **24,1** | **10,2** | **21,4** |
| Ir-SigP-252751 | Secreted metalloprotease | 0,0E+00 | 100 | **1,2** | **0,5** | **0,0** | **4,4** | **11,6** | **10,3** |
| Ir-SigP-278363 | peptidase family m13 | 0,0E+00 | 38,2 | **0,2** | **2,6** | **0,0** | **18,6** | **8,6** | **13,4** |
| Ir-SigP-245232 | SCP-like extracellular protein | 0,0E+00 | 100 | **1,5** | **1,0** | **0,4** | **12,9** | **7,1** | **13,0** |
| Ir-248185 | glucosylceramide beta-14-galactosyltransferase | 0,0E+00 | 78,8 | **1,2** | **2,4** | **0,4** | **16,3** | **15,0** | **10,6** |
| Ir-260093 | monocarboxylate transporter | 0,0E+00 | 100 | **4,2** | **1,8** | **1,6** | **34,5** | **15,9** | **27,7** |
| Ir-267946 | Aldehyde dehydrogenase | 0,0E+00 | 95,2 | **2,9** | **2,5** | **1,1** | **26,2** | **23,5** | **13,2** |
| Ir-265943 | Glutamate/aspartate and neutral amino acid transporters | 0,0E+00 | 90,3 | **7,5** | **8,6** | **2,7** | **76,5** | **32,0** | **63,9** |
| Ir-SigP-244280 | cytotoxin-like protein | 0,0E+00 | 97,1 | **31,1** | **3,8** | **54,9** | **337,8** | **133,0** | **221,6** |
| Ir-SigP-257511 | BTSP | 0,0E+00 | 95,7 | **385,4** | **155,2** | **213,5** | **1723,5** | **1010,7** | **2564,8** |
| Ir-259216 | mitochondrial/plastidial beta-ketoacyl-acp reductase | 0,0E+00 | 100 | **0,9** | **1,4** | **0,0** | **3,0** | **5,3** | **6,3** |
| Ir-237359 | monocarboxylate transporter | 0,0E+00 | 100 | **27,3** | **35,6** | **1,4** | **172,2** | **112,9** | **120,6** |
| Ir-SigP-258964 | ml domain protein | 0,0E+00 | 100 | **7,4** | **9,3** | **0,4** | **36,3** | **37,7** | **30,2** |
| Ir-254113 | choline transporter-like protein 1 | 0,0E+00 | 100 | **1,4** | **2,6** | **3,3** | **21,0** | **7,2** | **15,3** |
