# Supplementary material for: Sialome diversity of ticks revealed by RNAseq of single tick salivary glands
Source: PLoS Negl Trop Dis. 2018 Apr 13;12(4):e0006410. doi: 10.1371/journal.pntd.0006410 (PMC5919021; doi:10.1371/journal.pntd.0006410)
Supplement: S4 Table — Three independent libraries (1‒3) were used for each time-point. (DOCX) [file pntd.0006410.s005.docx]

**S4 Table.** **Overview of RKPM values for significantly up-regulated contigs in rabbit-fed (R) ticks fed for 72 h compared 48 h.** Three independent libraries (1‒3) were used for each time-point

| **Link to Pep** | **Comments** | **E value** | **Coverage %** | R48_1 RPKM | R48_2 RPKM | R48_3 RPKM | R72_1 RPKM | R72_2 RPKM | R72_3 RPKM |
| --- | --- | --- | --- | --- | --- | --- | --- | --- | --- |
| Ir-246087 | ixodes 10 kda peptide protein | 3,0E-62 | 84,3 | **0,0** | **0,0** | **1,3** | **184,1** | **122,0** | **67,3** |
| Ir-267037 | Plectin | 3,0E-60 | 30 | **0,1** | **0,0** | **0,0** | **1,2** | **1,4** | **1,1** |
| Ir-273314 | aquaporin major intrinsic protein family - 6 | 0,0E+00 | 100 | **4,7** | **2,0** | **21,5** | **177,8** | **148,1** | **118,0** |
| Ir-276739 | chymotrypsin inhibitor | 3E-58 | 100 | **13,7** | **1,2** | **9,2** | **76,7** | **169,1** | **83,5** |
| Ir-SigP-277286 | Sodium-coupled monocarboxylate transporter 2 | 0,0E+00 | 100 | **4,6** | **0,0** | **4,4** | **28,4** | **21,2** | **51,4** |
| Ir-SigP-263673 | peptidoglycan recognition protein | 2E-63 | 63,4 | **1,6** | **1,4** | **5,2** | **12,8** | **34,8** | **39,1** |
| Ir-SigP-500 | beta-16-n-acetylglucosaminyltransferase | 5E-93 | 100 | **7,7** | **19,3** | **1,5** | **102,3** | **98,2** | **79,9** |
| Ir-SigP-259801 | Transforming growth factor beta bone morphogenetic protein | 4,0E-14 | 85,4 | **0,4** | **0,2** | **1,1** | **6,1** | **2,5** | **5,6** |
| Ir-SigP-94 | 8.9 kda protein | 6,0E-38 | 100 | **47,1** | **23,9** | **113,4** | **418,6** | **330,3** | **470,5** |
| Ir-SigP-278363 | peptidase family m13 | 0,0E+00 | 38,2 | **18,6** | **8,6** | **13,4** | **64,3** | **127,6** | **71,9** |
| Ir-237908 | 8.9 kda protein | 2,0E-41 | 100 | **70,9** | **35,6** | **121,0** | **590,4** | **397,9** | **452,3** |
| Ir-274695 | IS4EU-1_NV | 0,0E+00 | 40,3 | **0,0** | **0,3** | **0,1** | **0,7** | **0,9** | **0,9** |
| Ir-SigP-257511 | BTSP | 4,0E-39 | 75 | **1723,5** | **1010,7** | **2564,8** | **6096,9** | **14223** | **7831,9** |
| Ir-SigP-6194 | Tyrosine phosphatase receptor type q isoform 1 precursor | 0,0E+00 | 97,6 | **1,0** | **0,3** | **0,3** | **2,9** | **2,7** | **3,3** |
| Ir-257508 | BTSP | 1,0E-36 | 67 | **2233,2** | **1318,2** | **3297,7** | **7545,8** | **17590** | **9604,9** |
