# Supplementary material for: Sialome diversity of ticks revealed by RNAseq of single tick salivary glands
Source: PLoS Negl Trop Dis. 2018 Apr 13;12(4):e0006410. doi: 10.1371/journal.pntd.0006410 (PMC5919021; doi:10.1371/journal.pntd.0006410)
Supplement: S5 Table — Three independent libraries (1‒3) were used. (DOCX) [file pntd.0006410.s006.docx]

**S5 Table.** **Overview of RKPM values for most abundant transcripts in 24 h libraries of naturally-fed ticks.** Three independent libraries (1‒3) were used.

| **Link to Pep** | **Comments** | **E value** | **Coverage %** | R24_1 RPKM | R24_2 RPKM | R24_3 RPKM |
| --- | --- | --- | --- | --- | --- | --- |
| Ir-SigP-242556 | 18.3 kda subfamily of the Basic tail superfamily | 2,00E-59 | 100 | **17011,5** | **6302,5** | **15365,9** |
| Ir-SigP-243468 | hypothetical secreted protein | 1000 | 50,7 | **9502,8** | **10193,1** | **7908,9** |
| Ir-267152 | hypothetical protein iscw_iscw017886 | 5E-26 | 80,2 | **2363,6** | **3468,1** | **5194,7** |
| Ir-240189 | translation elongation factor EF-1 alpha/Tu | 0 | 95,8 | **3126,0** | **2376,4** | **4671,9** |
| Ir-SigP-268393 | Glycine rich protein | 0 | 64 | **3198,6** | **3611,8** | **2378,2** |
| Ir-238256 | 60s acidic ribosomal protein p1 | 1E-61 | 100 | **2389,3** | **2114,8** | **4372,4** |
| Ir-239007 | 40S ribosomal protein | 0 | 107,8 | **2122,9** | **1973,8** | **3979,1** |
| Ir-253398 | salivary lipocalin | 2E-35 | 75,5 | **3279,1** | **1229,4** | **2102,5** |
| Ir-263963 | ribosomal protein large p2 | 4,00E-43 | 78 | **1599,4** | **1532,8** | **3296,6** |
| Ir-SigP-219629 | 18.3 kda subfamily of the Basic tail superfamily | 3E-55 | 99,3 | **2695,6** | **987,3** | **2594,7** |
| Ir-SigP-263812 | hypothetical secreted protein | 1,00E+03 | 52,3 | **1426,9** | **1258,9** | **2624,1** |
| Ir-SigP-243206 | salivary lipocalin | 0 | 97,7 | **2514,1** | **1093,7** | **1579,0** |
| Ir-243167 | ribosomal protein S25 | 3E-58 | 83,5 | **1474,4** | **1211,9** | **2484,2** |
| Ir-238412 | Secreted metalloprotease | 0 | 84,6 | **1747,5** | **2402,2** | **815,5** |
| Ir-261361 | ribosomal protein L13A | 6E-83 | 71,2 | **1433,4** | **1262,2** | **2249,6** |
