# Supplementary material for: Sialome diversity of ticks revealed by RNAseq of single tick salivary glands
Source: PLoS Negl Trop Dis. 2018 Apr 13;12(4):e0006410. doi: 10.1371/journal.pntd.0006410 (PMC5919021; doi:10.1371/journal.pntd.0006410)
Supplement: S6 Table — Three independent libraries (1‒3) were used. (DOCX) [file pntd.0006410.s007.docx]

**S6 Table.** **Overview of RKPM values for most abundant transcripts in 48 h libraries of naturally-fed ticks.** Three independent libraries (1‒3) were used.

| **Link to Pep** | **Comments** | **Evalue** | **Coverage %** | R48_1 RPKM | R48_2 RPKM | R48_3 RPKM |
| --- | --- | --- | --- | --- | --- | --- |
| Ir-SigP-271268 | E3 ubiquitin ligase partial - probable fragment | 9,00E-15 | 53,4 | **34701,5** | **37792,5** | **32299,7** |
| Ir-SigP-242380 | tick kunitz 56 | 4E-54 | 100 | **15966,9** | **9410,1** | **11859,6** |
| Ir-238412 | Secreted metalloprotease | 0 | 84,6 | **4155,9** | **2219,0** | **4371,7** |
| Ir-238410 | Secreted metalloprotease | 0 | 79,5 | **4078,8** | **2215,1** | **4088,7** |
| Ir-228908 | mitochondrial NADH-ubiquinone oxidoreductase chain 5 | 0 | 65,3 | **3730,9** | **2250,3** | **3587,0** |
| Ir-254303 | ribosomal protein L37A | 3E-30 | 83 | **2381,6** | **4156,5** | **2379,0** |
| Ir-238256 | 60s acidic ribosomal protein p1 | 1E-61 | 100 | **2586,3** | **3325,7** | **2433,1** |
| Ir-240189 | translation elongation factor EF-1 alpha/Tu | 0 | 95,8 | **2863,1** | **2701,4** | **2774,4** |
| Ir-257508 | BTSP | 1,00E-36 | 67 | **2233,2** | **1318,2** | **3297,7** |
| Ir-239007 | 40S ribosomal protein | 0 | 107,8 | **1916,5** | **2852,1** | **1797,9** |
| Ir-SigP-237291 | Secreted metalloprotease | 0,00E+00 | 63,7 | **2429,9** | **1005,4** | **2980,5** |
| Ir-255105 | BTSP | 0 | 66,6 | **1976,0** | **1507,3** | **2860,8** |
| Ir-255790 | metalloprotease partial | 0 | 78,6 | **2709,1** | **2309,0** | **1289,5** |
| Ir-SigP-268393 | Glycine rich protein | 0 | 64 | **1911,3** | **1851,3** | **2324,0** |
| Ir-SigP-259375 | Secreted metalloprotease | 3E-77 | 94,7 | **2248,5** | **2341,5** | **1306,7** |
