# Supplementary material for: Sialome diversity of ticks revealed by RNAseq of single tick salivary glands
Source: PLoS Negl Trop Dis. 2018 Apr 13;12(4):e0006410. doi: 10.1371/journal.pntd.0006410 (PMC5919021; doi:10.1371/journal.pntd.0006410)
Supplement: S7 Table — Three independent libraries (1‒3) were used. (DOCX) [file pntd.0006410.s008.docx]

**S7 Table.** **Overview of RKPM values for most abundant transcripts in 72 h libraries of naturally-fed ticks.** Three independent libraries (1‒3) were used.

#

| **Link to Pep** | **Comments** | **E value** | **Coverage %** | R72_1 RPKM | R72_2 RPKM | R72_3 RPKM |
| --- | --- | --- | --- | --- | --- | --- |
| Ir-SigP-271268 | E3 ubiquitin ligase partial - probable fragment | 9,00E-15 | 53,4 | **36378,9** | **27275,6** | **37353,6** |
| Ir-263609 | BTSP | 7E-41 | 73,3 | **12594,1** | **19351,0** | **16477,1** |
| Ir-263610 | BTSP | 1E-35 | 65,8 | **11324,8** | **17495,9** | **13704,1** |
| Ir-257508 | BTSP | 1E-36 | 67 | **7545,8** | **17590,2** | **9604,9** |
| Ir-SigP-264569 | BTSP | 0 | 75 | **6483,8** | **15543,2** | **11660,5** |
| Ir-SigP-257511 | BTSP | 4E-39 | 75 | **6096,9** | **14223,3** | **7831,9** |
| Ir-238412 | Secreted metalloprotease | 0 | 84,6 | **4860,1** | **7337,6** | **3660,0** |
| Ir-228908 | mitochondrial NADH-ubiquinone oxidoreductase chain 5 | 2E-21 | 65,3 | **6455,3** | **2996,5** | **5718,9** |
| Ir-238410 | Secreted metalloprotease | 0,00E+00 | 79,5 | **4717,8** | **6801,3** | **3405,2** |
| Ir-SigP-263611 | BTSP | 3E-39 | 55 | **3074,4** | **3957,9** | **6320,8** |
| Ir-266381 | mitochondrial f1f0-atp synthase subunit c/atp9/proteolipid | 1,00E-77 | 100 | **2861,1** | **3430,8** | **2812,4** |
| Ir-240189 | translation elongation factor EF-1 alpha/Tu | 0 | 95,8 | **2683,3** | **3194,2** | **2897,5** |
| Ir-266382 | ATP synthase c-subunit | 1E-47 | 74,3 | **2686,3** | **3110,7** | **2528,1** |
| Ir-254303 | ribosomal protein L37A | 0 | 83 | **2251,9** | **2920,0** | **2329,4** |
| Ir-236814 | sodium/potassium-transporting atpase subunit beta | 0 | 91,1 | **2276,1** | **2860,9** | **2322,3** |
