# Supplementary material for: Sialome diversity of ticks revealed by RNAseq of single tick salivary glands
Source: PLoS Negl Trop Dis. 2018 Apr 13;12(4):e0006410. doi: 10.1371/journal.pntd.0006410 (PMC5919021; doi:10.1371/journal.pntd.0006410)
Supplement: S8 Table — Three independent libraries (1‒3) were used for each mode of feeding. Transcripts were listed by statistical significance and were filtered with coverage > 50, average RPKM > 10, and fold change > 5. (DOCX) [file pntd.0006410.s009.docx]

# S8 Table. Overview of RKPM values for significantly up-regulated contigs in rabbit-fed (R) compared to membrane-fed (M) ticks fed for 48 hours. Three independent libraries (1‒3) were used for each mode of feeding. Transcripts were listed by statistical significance and were filtered with coverage > 50, average RPKM > 10, and fold change > 5.

| **Link to Pep** | **Comments** | **E value** | **Coverage %** | M48_1 RPKM | M48_2 RPKM | M48_3 RPKM | R48_1 RPKM | R48_2 RPKM | R48_3 RPKM |
| --- | --- | --- | --- | --- | --- | --- | --- | --- | --- |
| Ir-267022 | hypothetical protein iscw_iscw019487 | 4E-40 | 100 | **3,1** | **1,8** | **1,3** | **12,9** | **14,2** | **9,5** |
| Ir-SigP-242380 | tick kunitz 56 | 4,00E-54 | 100 | **3,4** | **8,9** | **2,6** | **15966,9** | **9410,1** | **11859,6** |
| Ir-243419 | tetratricopeptide repeat protein 8 | 8E-43 | 93,3 | **3,8** | **2,0** | **2,0** | **23,6** | **13,8** | **27,3** |
| Ir-263857 | tick transposon | 6,00E-38 | 100 | **0,1** | **0,2** | **0,4** | **15,0** | **6,8** | **9,3** |
| Ir-SigP-244872 | serine protease with signal anchor | 0,00E+00 | 100 | **3,7** | **7,1** | **4,4** | **25,4** | **35,4** | **17,1** |
| Ir-SigP-252751 | hypothetical secreted protein precursor | 1,00E+03 | 52,6 | **1,1** | **2,8** | **2,9** | **7,2** | **15,9** | **17,8** |
| Ir-SigP-248779 | cytotoxin-like protein | 0 | 100 | **0,7** | **0,5** | **13,8** | **54,1** | **134,8** | **69,5** |
| Ir-242974 | cytotoxin-like protein partial | 0,00E+00 | 88,7 | **2,6** | **1,1** | **21,5** | **96,8** | **243,2** | **111,7** |
