# Supplementary material for: Sialome diversity of ticks revealed by RNAseq of single tick salivary glands
Source: PLoS Negl Trop Dis. 2018 Apr 13;12(4):e0006410. doi: 10.1371/journal.pntd.0006410 (PMC5919021; doi:10.1371/journal.pntd.0006410)
Supplement: S9 Table — Three independent libraries (1‒3) were used for each mode of feeding. Transcripts were listed by statistical significance and were filtered with coverage > 50, average RPKM > 10, and fold change > 5. (DOCX) [file pntd.0006410.s010.docx]

# S9 Table. Overview of RKPM values for significantly up-regulated contigs in rabbit-fed (R) compared to membrane-fed (M) ticks fed for 72 hours. Three independent libraries (1‒3) were used for each mode of feeding. Transcripts were listed by statistical significance and were filtered with coverage > 50, average RPKM > 10, and fold change > 5.

| **Link to Pep** | **Comments** | **E value** | **Coverage %** | M72_1 RPKM | M72_2 RPKM | M72_3 RPKM | R72_1 RPKM | R72_2 RPKM | R72_3 RPKM |
| --- | --- | --- | --- | --- | --- | --- | --- | --- | --- |
| Ir-242985 | 5'-nucleotidase | 0,0E+00 | 93,5 | **91,0** | **57,7** | **59,4** | **424,8** | **405,9** | **362,4** |
| Ir-SigP-94 | 8.9 kda protein | 6,0E-38 | 100 | **92,6** | **8,9** | **50,9** | **418,6** | **330,3** | **470,5** |
| Ir-SigP-500 | beta-16-n-acetylglucosaminyltransferase | 5,0E-93 | 100 | **22,9** | **26,3** | **0,0** | **102,3** | **98,2** | **79,9** |
| Ir-260891 | Secreted metalloprotease | 0,0E+00 | 71,2 | **0,5** | **11,6** | **1,7** | **29,1** | **34,4** | **35,2** |
| Ir-240649 | reverse transcriptase | 0,0E+00 | 93 | **12,7** | **2,8** | **13,6** | **58,8** | **40,2** | **55,3** |
| Ir-237989 | mitochondrial/plastidial beta-ketoacyl-acp reductase | 0,0E+00 | 71,6 | **7,0** | **0,1** | **1,1** | **639,2** | **1156,6** | **1308,2** |
| Ir-245862 | acireductone dioxygenase | 3,0E-87 | 100 | **1,7** | **3,9** | **2,2** | **30,0** | **15,6** | **26,5** |
| Ir-248185 | glucosylceramide beta-14-galactosyltransferase | 0,0E+00 | 78,8 | **11,1** | **8,3** | **4,2** | **35,6** | **70,8** | **46,0** |
| Ir-258748 | pancreatic lipase-like enzyme partial | 5,0E-77 | 100 | **3,1** | **2,5** | **0,1** | **8,3** | **8,1** | **13,8** |
| Ir-SigP-252751 | hypothetical secreted protein precursor | 1,0E+03 | 52,6 | **1,9** | **3,4** | **2,2** | **73,7** | **36,6** | **37,4** |
| Ir-SigP-277286 | Sodium-coupled monocarboxylate transporter 2 | 0,0E+00 | 100 | **0,4** | **0,7** | **0,3** | **28,4** | **21,2** | **51,4** |
| Ir-246087 | ixodes 10 kda peptide protein | 3,0E-62 | 84,3 | **5,3** | **1,8** | **0,0** | **184,1** | **122,0** | **67,3** |
| Ir-SigP-241910 | beta-16-n-acetylglucosaminyltransferase | 2,0E-89 | 100 | **31,3** | **37,3** | **0,1** | **75,9** | **163,0** | **122,6** |
| Ir-19983 | salivary lipocalin | 1,0E-25 | 79 | **0,0** | **0,7** | **3,2** | **1115,3** | **455,8** | **549,2** |
| Ir-SigP-264966 | salivary kunitz domain protein | 4,0E-60 | 101,6 | **3,6** | **8,0** | **3,4** | **32,2** | **92,1** | **92,1** |
| Ir-240961 | ixoderin B5 | 0,0E+00 | 92,3 | **89,3** | **31,7** | **136,8** | **745,9** | **456,8** | **325,6** |
| Ir-SigP-243736 | phospholipase partial | 0,0E+00 | 75 | **1,0** | **3,1** | **0,0** | **11,6** | **7,7** | **19,6** |
| Ir-SigP-263664 | hypothetical secreted protein precursor | 1,0E+03 | 54,3 | **0,1** | **0,7** | **18,3** | **36,0** | **98,1** | **66,5** |
| Ir-SigP-252751 | Secreted metalloprotease | 0,0E+00 | 100 | **0,8** | **1,0** | **0,6** | **50,9** | **19,8** | **22,7** |
| Ir-SigP-268857 | cytotoxin-like protein | 0,0E+00 | 98,5 | **9,2** | **40,5** | **12,1** | **165,2** | **62,7** | **113,7** |
| Ir-237699 | 5'-nucleotidase | 0,0E+00 | 79 | **95,1** | **60,2** | **104,7** | **221,7** | **687,7** | **583,8** |
